# Upfront Taxane Could Be Superior to Pegylated Liposomal Doxorubicin (PLD): A Retrospective Real-World Analysis of Treatment Sequence Taxane–PLD versus PLD–Taxane in Patients with Metastatic Breast Cancer

**DOI:** 10.3390/cancers15204953

**Published:** 2023-10-12

**Authors:** Till Wallrabenstein, Anton Oseledchyk, Eveline Daetwyler, Christoph Rochlitz, Marcus Vetter

**Affiliations:** 1 University Hospital Basel, Medical Oncology, Petersgraben 4, 4031 Basel, Switzerlandmarcus.vetter@ksbl.ch (M.V.); 2 University Medical Center Freiburg, Hematology and Oncology, Hugstetter Strasse 55, 79106 Freiburg, Germany; 3 Zentrum Onkologie & Hämatologie, Tumorzentrum, Kantonsspital Baselland, Rheinstrasse 26, 4410 Liestal, Switzerland; 4 Medical Faculty, University Basel, 4031 Basel, Switzerland

**Keywords:** metastatic breast cancer, real-world data, treatment sequence, pegylated liposomal doxorubicin, taxane

## Abstract

**Simple Summary:**

Patients with breast cancer, that has spread and does not respond to hormone treatment, are usually treated with chemotherapy. If one kind of chemotherapy fails, another kind is started. There are various options and different chemotherapies to choose, but currently there is no standard, which kind to use first. Our aim was to find out, whether starting with one chemotherapy is better than with the other. We have investigated all patients with metastatic breast cancer who have been treated with the two most frequently used kinds of chemotherapy (“taxanes” and “PLD”) at the University Hospital Basel, Switzerland. We found that patients who have received taxanes first survived longer than patients who received PLD first. These results are astonishing and highly relevant to patients with breast cancer and their treating physicians. Because our study was backward-looking, we cannot rule out that our results are confounded by any unknown factors other than the kind of chemotherapy. This important question should be investigated in a prospective clinical trial.

**Abstract:**

**Background:** Patients with endocrine-resistant metastatic breast cancer (MBC) require cytostatic therapy. Single-agent taxanes and anthracyclines, including pegylated liposomal doxorubicin (PLD), are standard treatment options. There are no prospective data regarding optimal treatment sequences, and real-world data regarding both treatment options are limited. **Methods:** We analyzed electronic records of all patients with Her2-negative MBC treated with either first-line PLD or first-line taxane and subsequent crossover at the University Hospital Basel between 2003 and 2021. The primary endpoint was time to next chemotherapy or death (TTNC). Secondary endpoints were overall survival (OS), progression-free survival (PFS), and objective response rate (ORR). We used the Kaplan–Meyer method and logrank test to compare time-to-event endpoints and the Fisher exact test to compare discrete variables. **Results:** We retrospectively identified 42 patients with Her2-negative MBC who have received either single-agent PLD or single-agent taxane as first-line chemotherapy with subsequent crossover, including 23 patients who received first-line PLD and 19 patients who received first-line taxane. Baseline characteristics were similar between treatment groups. Treatment sequence PLD–taxane was significantly inferior to taxane–PLD regarding all endpoints: median TTNC 4.9 vs. 9.9 months (*p* = 0.006), median OS 17.8 vs. 24.6 months (*p* = 0.05), median PFS 4.4 vs. 9.0 months (*p* = 0.005), and ORR 13% vs. 53% (*p* = 0.01). **Conclusions:** Here, we report a first retrospective head-to-head comparison of the treatment sequence PLD–taxane versus taxane–PLD in patients with MBC, showing a substantial advantage of using taxanes first, followed by PLD. An inherent treatment bias in favor of first-line taxanes cannot be excluded, thus calling for prospective validation.

## 1. Background

Breast cancer (BC) is a major global healthcare burden. It is the most frequently diagnosed cancer among women and the second most frequent cancer-related cause of death among women in the United States [1]. Worldwide, an estimated 2.3 million new breast cancer cases occurred in 2020, and 0.7 million deaths were attributable to BC [2]. About 20–30% of patients with localized BC develop metastatic relapse at a later time [3].

Endocrine-resistant Her-2-negative breast cancer is usually treated with conventional chemotherapy, although new antibody–drug conjugates, including trastuzumab deruxtecan and Sacituzumab govitecan, are changing the treatment landscape [4,5]. Current guidelines recommend sequential single-agent chemotherapy, adequate options of which include anthracyclines, taxanes, capecitabine, gemcitabine, and microtubule inhibitors such as vinorelbine and eribulin [6,7,8]. Combination therapies are usually reserved for fitter patients with visceral disease, a high tumor burden, and/or a high need for response. Bevacizumab, in combination with paclitaxel, has been associated with a higher response rate in the first line but no overall survival benefit [9]. Guidelines, including NCCN, AGO, and ESMO, and prospective evidence do not clearly favor one regimen over another. In clinical practice, most eligible patients are treated with either single-agent taxane (paclitaxel or docetaxel) or pegylated liposomal doxorubicin (PLD) in the first line and cross over to the other in the second or third line. Other options, such as single-agent capecitabine or vinorelbine, are less frequently used in the first-line setting. This analysis was developed as part of a larger project aiming to analyze different treatment sequences in MBC. There is no previous evidence (neither trial data nor real-world data) regarding the optimal treatment sequence in MBC, which is an unmet need in clinical practice. Given that single-agent taxanes (paclitaxel/docetaxel) and PLD were the two most frequently used cytostatic agents in first-line chemotherapy at our center, we decided to focus on these agents, also because there is no direct prospective evidence comparing these two options. Our hypothesis was that up-front PLD is equally effective as up-front taxane treatment in patients who are eligible for both options.

PLD has become a preferred alternative to conventional anthracyclines, especially for patients with increased cardiac risk, previous exposure to conventional anthracyclines, and the elderly. PLD was found to have a similar efficacy and a lower rate of cardiac events, alopecia, nausea, and myelosuppression than single-agent conventional doxorubicin, but a higher rate of palmar–plantar erythrodysesthesia (PPE) [10]. There are specific data in elderly patients with MBC, including a randomized phase III trial, suggesting that PLD has a very good safety profile and is as effective as single-agent capecitabine [11]. Available evidence comparing PLD to other treatment options in the setting of first or second-line treatment is summarized in Table 1. However, there are no trials that have directly compared PLD and taxanes regarding efficacy and/or patient-related outcome measures.

No randomized prospective trial has investigated taxanes (paclitaxel or docetaxel) versus PLD in the first-line setting. There is only indirect evidence for comparable efficacy based on the pivotal E1193-trial published by Sledge et al., which has compared conventional doxorubicin, paclitaxel, and the combination of both agents as first-line chemotherapy for MBC, finding no significant difference in median time to treatment failure and OS for patients treated with either of these options [14].

Direct comparative evidence of first-line PLD versus taxane (paclitaxel or docetaxel) treatment in patients with MBC who are eligible for both options is desirable and answers a relevant question in daily clinical practice. Therefore, our aim was a retrospective head-to-head comparison of first-line PLD versus paclitaxel/docetaxel in a real-world group of patients with endocrine-resistant advanced HER2-negative breast cancer.

## 2. Material and Methods

### 2.1. Study Design and Patient Population

We conducted a keyword search of electronic hospital records to identify all patients with histologically confirmed Her-2-negative MBC (irrespective of HER2-low status) treated with either first-line PLD (group ‘PLD’) or first-line taxane (group ‘TAX’) and subsequent reciprocal crossover at the University Hospital Basel, Switzerland, between 1 July 2003, and 31 May 2021. Crossover was mandatory to avoid selection bias by ensuring that all patients were generally eligible for both treatment options. All patients received 40 mg/m^2^ of PLD (“Caelyx,” Baxter), administered intravenously every 4 weeks. Taxane treatment consisted of weekly intravenous paclitaxel (80 mg/m^2^, d1/8/15, q4w) in most cases. However, two patients in each group have received intravenous docetaxel (75 mg/m^2^, q3w). We included patients who have received previous endocrine therapy and/or CDK4/6 inhibitors to treat MBC before first-line chemotherapy. Patient charts were viewed, and relevant data points were manually retrieved from an anonymized database. Ethics approval for this study was granted by the responsible ethics committee, Ethikkommission Nordwest-und Zentralschweiz (EKNZ, Basel, Switzerland), on 21 July 2021 (project ID 2021-00709) and included a waiver for informed consent regarding the use of health-related data by patients unable to provide consent (e.g., deceased patients).

### 2.2. Aims and Endpoints

The primary endpoint of this study was time to next chemotherapy or death (TTNC), defined as the time from treatment initiation until the start of a subsequent line of chemotherapy or death. TTNC was selected as a clinically meaningful endpoint because treatment failures for reasons other than progression (e.g., clinical deterioration, toxicity, or patient preference) are implicated, even if such data points are missing due to the retrospective nature of this study. Secondary endpoints were overall survival (OS), progression-free survival after initiation of first line chemotherapy (PFS), and objective response rate (ORR). OS was defined as the time from treatment initiation until death or loss of follow-up. PFS was defined as the time from treatment initiation until radiographic progression, death, or loss of follow-up. Regarding all time-to-event endpoints, patients who were lost to follow-up were censored at the time of last contact. Response evaluation was taken from routine CT scans and was defined according to RECIST 1.1 criteria [15]. If RECIST criteria were unavailable for response evaluation in single patients due to the retrospective nature of our study, no progression was documented unless unequivocal clinical progression had been recorded by the treating physician (e.g., new cutaneous metastasis). The last update for time-dependent variables was on 31 January 2022.

### 2.3. Statistical Analysis

Our data were not pre-processed. Baseline and treatment characteristics are presented descriptively and were compared by using Fisher’s exact test for qualitative e-variables and the two-tailed Mann–Whitney U test for quantitative variables, hypothesizing that there was no difference between groups. We analyzed time-to-event endpoints by the Kaplan–Meyer method and tested for significance by the logrank test, hypothesizing that there was no difference between groups. Confidence intervals for time-to-event endpoints and categorical endpoints were calculated by given z-values and by the Clopper–Pearson exact method, respectively. The significance level was defined as *p* < 0.05 without correction for multiple testing. We used SPSS version 28 (IBM Corp., Chicago, IL, USA) for all statistical analyses.

## 3. Results

### 3.1. Patients

We retrospectively identified 42 patients with Her2-negative MBC who had received either single-agent PLD or taxane (paclitaxel or docetaxel) as first-line chemotherapy between July 2003 and May 2021 at the University Hospital Basel. A total of 23 patients (group ‘PLD’) have received first-line PLD and later taxane (21 patients received weekly paclitaxel and 2 patients received docetaxel). A total of 19 patients (group ‘TAX’) have received first-line taxane (17 patients received weekly paclitaxel and 2 patients received docetaxel) and later PLD. There was no significant difference between groups regarding age at treatment initiation, comorbidities, disease biology (histology, grade, receptor status), tumor stage at the time of diagnosis, distribution of metastases, or previous operative/neoadjuvant/adjuvant/radiotherapeutic treatment (Table 2). About two-thirds of patients in both groups have received previous endocrine therapy for MBC before first-line chemotherapy. Patients in both groups have received a median of five treatment lines in total (including endocrine treatments) and a median of three subsequent lines of chemotherapy after first-line PLD/taxane.

### 3.2. Outcome Parameters

**Figure 1:**
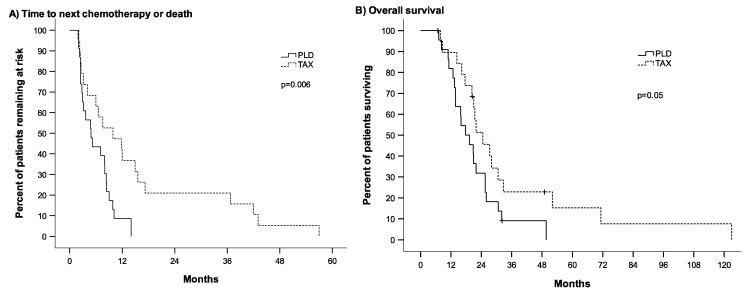
Time to initiation of next chemotherapy or death (**A**) and overall survival (**B**) in 42 patients with Her-2-negative metastatic breast cancer who had received either single-agent pegylated liposomal doxorubicin (PLD) or single-agent taxane in first-line chemotherapy with subsequent crossover. Patients in group PLD (n = 23) have received first-line PLD and later taxane. Patients in group TAX (n = 19) have received first-line taxane and later PLD. *p* = *p*-value. TAX = taxane.

The median TTNC of patients treated with first-line PLD was 4.9 months (95% CI 2.5–7.3) compared to 9.9 months (95% CI 2.3–17.5) in patients treated with first-line taxane, *p* = 0.006 (Figure 1A). Median OS after initiation of first-line chemotherapy with PLD was 17.8 months (95% CI 12.2–23.5) and 24.6 months (95% CI 16.7–32.6) with first-line taxane, *p* = 0.05 (Figure 1B). The median PFS of patients receiving first-line PLD was 4.4 months (95% CI 0.93–7.8) as compared to 9.0 months (95% CI 5.4–12.7) in patients receiving first-line taxane, *p* = 0.005. All patients in group PLD were evaluable for response, and four patients in group TAX were non-evaluable due to missing data. There was no complete remission in either group. ORR was 13% in group PLD as compared to 53% in group TAX, *p* = 0.01. The clinical benefit rate did not differ significantly between groups (52% in group PLD, 73% in group TAX, *p* = 0.2).

As stated before, group TAX included 17 patients who had received weekly paclitaxel (80 mg on days 1/8/15, q4w) and 2 patients who had received docetaxel (75 mg, q3w). To rule out that our results were biased using two different taxanes, we have performed an additional analysis, comparing paclitaxel to PLD and docetaxel to PLD separately. TTNC in patients treated with first-line paclitaxel was 9.9 months as compared to 4.9 months in group PLD (*p* = 0.007), OS was 22 months as compared to 17.8 months in group PLD (*p* = 0.05), and ORR was 67% as compared to 13% in group PLD (*p* = 0.001). TTNC in patients treated with first-line docetaxel was 8 months (*p* = 0.58 in comparison to PLD), OS was 24.6 months (*p* = 0.68 in comparison to PLD), and ORR was 50% (*p* = 0.3 in comparison to PLD). The group of patients having received first-line docetaxel was too small (n = 2) to render statistically significant results in comparison to PLD. However, the results were generally comparable to those accomplished with paclitaxel. 

### 3.3. Subsequent Treatments and Crossover

One patient in group PLD has received interim endocrine treatment before second-line chemotherapy, as compared to 8 patients (42%) in group TAX (*p* = 0.006). Patients in group TAX have received PLD in the second line (9 patients), third line (5 patients), or later lines (5 patients). All patients in group PLD have received taxane in the second line (20 patients) or third line (3 patients). Median TTNC2 and PFS2 from subsequent PLD in group TAX were 3.4 months and 3.8 months, respectively. In comparison, the median TTNC2 and PFS2 from subsequent taxane in group PLD were 5.8 months and 5.3 months, respectively. ORR for PLD after the previous taxane was 17%, compared to an ORR of 20% for taxane after the previous PLD. We did not test for significance regarding crossover results because this comparison was not a pre-planned analysis and because groups were too heterogeneous regarding treatment lines.

## 4. Discussion

There is no consensus or previous evidence regarding the optimal sequencing of single-agent chemotherapy in patients with endocrine-resistant MBC. Commonly used agents in the first-line setting are taxanes (paclitaxel or docetaxel) and anthracyclines, including PLD. Our data presented here suggest a better outcome with first-line taxane than with first-line PLD.

The only prospective evidence comparing first-line taxane to first-line anthracycline treatment in patients with MBC has compared paclitaxel to conventional doxorubicin. Doxorubicin was equivalent to paclitaxel regarding time to treatment failure and ORR in this trial, and OS was insignificantly shorter (18.9 vs. 22.2 months). PLD has not been directly compared to taxane treatment. However, the equivalence of PLD to conventional doxorubicin was established in the first-line setting by O’Brian et al. in 2003. The trial was designed to test for non-inferiority and found comparable results for PLD and doxorubicin regarding PFS (median 6.9 vs. 7.8 months) and OS (median 21 vs. 22 months). Overall survival in our cohort was comparable to the results of phase III randomized controlled trials; however, it was not generally overwhelming and demonstrated a poor outcome for patients once endocrine therapy had been exhausted. More favorable outcomes can be assumed for current and future patients due to new treatment options, including CDK4/6 inhibitors, ADCs, and immunotherapy.

Weekly paclitaxel was the most frequently used taxane regimen in our patient group, which might explain the better outcome of upfront taxane treatment due to the known anti-angiogenic effects of weekly paclitaxel [16,17]. It might be that patients keep benefiting from the anti-angiogenic effect even after treatment discontinuation and after the initiation of second-line therapy. It might also be that the weekly dosing schedule of paclitaxel facilitates better treatment adherence, tolerability, and patient care, so patients are in better shape when they start second-line treatment. Finally, there might be other unknown underlying pathomechanisms of chemoresistance that render cancer cells more susceptible to PLD after previous taxane treatment but not vice versa. Also, the predictive role of disease biology and histology, e.g., in rare histological subtypes such as invasive micropapillary carcinoma of the breast, remains unclear with regard to chemosensitivity [18,19].

This study has various limitations due to its retrospective design. Data regarding dosing, toxicity, performance status, and patient-reported outcomes were not available for systematic analysis. The number of patients is relatively small. The results were significant; however, a larger sample size would have allowed subgroup analysis to determine which subpopulations of patients might have a greater benefit from which respective treatment option (e.g., patients with cardiac disease, histological subtypes, etc.). A proper comparison of endpoints from second-line treatment (OS2, PFS2, etc.) would have been desirable but was obscured by substantial heterogeneity between and within groups because not all patients have crossed over to PLD/taxane immediately.

Despite balanced baseline characteristics as provided in Table 2, a certain intergroup heterogeneity is likely, and there might be unknown confounders. From a clinical standpoint, it can be suspected that upfront taxane treatment is more likely chosen as first-line treatment in fitter patients, and PLD is more likely in less fit patients. PLD treatment was frequently postponed to third or later treatment lines in patients who had received first-line taxane. On the other hand, nearly all patients who have received first-line PLD have received taxane treatment immediately after the failure of PLD. These observations suggest that taxane treatment might have been perceived as a more potent treatment option than PLD. Although there is published data in support of PLD re-exposure, no patient has been rechallenged with PLD in our population, which is not surprising given the low response rate and short time-to-event endpoints observed here [20]. On the other hand, two patients who had received first-line paclitaxel or docetaxel were re-exposed to taxanes at a later time and given docetaxel/paclitaxel, respectively. Interestingly, patients starting with taxane chemotherapy were also more likely to receive interim endocrine treatment than patients receiving PLD in the first line, supporting again the assumption of a higher level of trust in taxane treatment. In consequence, an implicit treatment bias could contribute to the differing results between sequence options PLD → TAX and TAX → PLD, as observed here. Alternatively, up-front taxane treatment might be simply more effective than PLD. This question cannot finally be answered retrospectively, thus calling for prospective validation.

## 5. Conclusions

This is the first retrospective head-to-head comparison of first-line PLD vs. taxane treatment in patients with MBC, showing substantial advantages of using taxane first, followed by PLD, compared to the opposite sequence. Due to its retrospective design, this exploratory analysis has methodical limitations, and prospective validation is highly desirable.

## Figures and Tables

**Table 1:** Overview of relevant phase III trials investigating pegylated liposomal doxorubicin (PLD) in patients with metastatic breast cancer.

	O’Brien (2003) [10]	Harbeck (2017) [12]	Smorenburg (2013) [11]	Keller (2004) [13]
*Number of patients (PLD)*	254	105	40	150
*Control arm*	doxorubicin	capecitabine	capecitabine	vinorelbin/MV
*Treatment line*	first line	first line	first	second or later
**Inclusion criteria**				
- Performance status	ECOG ≤ 2	ECOG ≤ 2	ECOG ≤ 2	Karnofsky ≥ 60%
- Age (years)	>18	>18	≥65	>18
- Type	All	All	All	taxane-refractory
**Patient criteria**				
- Median age	59	62	75	56
- ≥3 sites of metastasis (%)	30	--	--	26
- Her2-positive (%)	--	--	2	--
- HR-positive (%)	35	--	62	47
- Visceral disease (%)	59	--	--	63
**Endocrine therapy (%)**	--	--	--	65
- adjuvant	--	--	48	54
- in MBC	--	--	60	64
***Prior chemotherapy (%)***	--	--	12	100
- thereof adjuvant			12	78
- in MBC			0	96
*Dosing (mg/m^2^)*	50, q4w	50, q4w	45, q4w	50, q4w
**Outcome measure**				
- ORR	33%	11%	18%	10%
- CBR	58%	--	72%	38%
- PFS (months)	6.9	6	5.6	2.9
- OS (months)	21	23.3	13.8	10.4

CBR = clinical benefit rate; ECOG = eastern cooperative oncology group performance status; Her-2 = human epidermal growth factor receptor 2; HR = hormone receptor; m^2^ = square meter; mg = milligram; MV = mitomycin-c and vinblastine; q4w = every 4 weeks.

**Table 2:** Baseline-, disease-, and treatment characteristics of 42 patients with Her-2-negative metastatic breast cancer had received either single-agent pegylated liposomal doxorubicin (PLD) or single-agent taxane in first-line chemotherapy with subsequent crossover.

Characteristic	First Line PLD (n = 23)	First Line Taxane (n = 19)	*p*
**Median age at treatment initiation**	61.5	59.8	0.74
**Breast cancer type**			
Luminal A *(%)*	8 *(35)*	6 *(32)*	1
Luminal B *(%)*	12 *(52)*	11 *(58)*	0.76
Triple-negative breast cancer *(%)*	3 *(13)*	2 *(11)*	1
**Receptor Status**			
ER-positive *(%)*	20 *(87)*	17 *(89)*	1
PR-positive *(%)*	18 *(78)*	15 *(79)*	1
**M-stage at primary diagnosis**			
M0/cMx *(%)*	18 *(78)*	16 *(84)*	0.71
M1 *(%)*	5 *(22)*	3 *(16)*	
**Relevant comorbidity**			
Heart/Cardiovascular *(%)*	2 *(9)*	1 *(5)*	1
Pulmonary *(%)*	3 *(13)*	0 *(0)*	0.24
Diabetes *(%)*	3 *(13)*	2 *(11)*	1
Arterial hypertension *(%)*	6 *(26)*	7 *(37)*	0.52
Other *(%)*	8 *(35)*	3 *(16)*	0.29
**Metastases at diagnosis of MBC**			
Bone *(%)*	17 *(74)*	10 *(53)*	0.11
Lung *(%)*	9 *(39)*	5 *(26)*	0.51
Liver *(%)*	5 *(22)*	6 *(32)*	0.50
Pleural *(%)*	2 *(9)*	2 *(11)*	1
CNS *(%)*	1 *(4)*	1 *(5)*	1
Peritoneal/abdominal *(%)*	2 *(9)*	2 *(11)*	1
Other sites *(%)*	11 *(48)*	11 *(58)*	0.55
Visceral disease *(%)*	12 *(52)*	10 *(53)*	1
1–2 organs involved *(%)*	15 *(65)*	12 *(63)*	1
≥3 organs involved *(%)*	8 *(35)*	7 *(37)*	--
**Primary treatment**			
Breast conservative surgery *(%)*	12 *(52)*	11 *(58)*	0.76
Breast ablative surgery *(%)*	6 *(26)*	7 *(37)*	0.52
Sentinel node resection *(%)*	10 *(43)*	7 *(37)*	0.76
Axillar revision *(%)*	11 *(48)*	10 *(53)*	1
**Neoadjuvant chemotherapy**			
Any *(%)*	1 *(4)*	3 *(16)*	0.31
Neoadjuvant taxanes *(%)*	1 *(4)*	2 *(11)*	0.58
Neoadjuvant anthracycline *(%)*	1 *(4)*	3 *(16)*	0.31
**Adjuvant chemotherapy**			
Any *(%)*	7 *(30)*	10 *(53)*	0.21
Taxane *(%)*	3 *(13)*	4 *(21)*	0.68
Anthracycline *(%)*	6 *(26)*	8 *(42)*	0.33
**Adjuvant endocrine therapy**			
Any *(%)*	13 *(57)*	14 *(74)*	0.34
Minimum of 5 years *(%)*	7 *(30)*	8 *(42)*	0.52
**Radiotherapy**			
Adjuvant RT *(%)*	14 *(61)*	16 *(84)*	0.17
RT for MBC before first line *(%)*	14 *(61)*	15 *(79)*	0.32
**Systemic treatment for MBC**			
Endocrine therapy before first CT *(%)*	17 *(74)*	13 *(68)*	0.74
cdk4/6 inhibitor before first CT *(%)*	9 *(39)*	4 *(21)*	0.32
Choice of taxane: paclitaxel weekly *(%)*	21 *(91)*	17 *(89)*	
Choice of taxane: docetaxel q3w *(%)*	2 *(9)*	2 *(11)*	1
Median endocrine lines before first CT	2	1	0.08
Median total treatment lines	5	5	0.84
Median subsequent treatment lines	3	3	0.36
Bisphosphonate/denosumab *(%)*	15 *(65)*	11 *(58)*	0.75

BC = breast cancer; CNS = central nervous system; CT = chemotherapy; ER = estrogen receptor; Her-2 = human epidermal growth factor receptor 2; MBC = metastatic breast cancer; n = number; *p* = *p*-value; PLD = pegylated liposomal doxorubicin; PR = progesterone receptor; n = number; RT = radiotherapy.

## Data Availability

The data that support the findings of this study are available upon reasonable request from the corresponding author. They cannot be made publicly available due to ethical restrictions regarding patient privacy.

## Abbreviations

BC	Breast cancer
CBR	Clinical benefit rate
Cdk4/6	Cyclin-dependent kinase 4/6
ECOG PS	Eastern Cooperative Oncology Group Performance Status
ER	Estrogen receptor
Her2	Human epidermal growth factor receptor 2
HR	Hazard ratio
MBC	Metastatic breast cancer
ORR	Objective response rate
OS	Overall Survival
PD-L1	Programmed cell death 1 ligand 1
PFS	Progression-free survival
PLD	Pegylated liposomal doxorubicin
PPE	Palmar–plantar erythrodysesthesia
PR	Progesterone receptor
SD	Stable disease
SERD	Selective estrogen receptor degrader
TNBC	Triple-negative breast cancer
TTNC	Time to next chemotherapy
